# The management of achondroplasia in Italy: results from a Delphi panel based on real-world experience

**DOI:** 10.3389/fped.2023.1209994

**Published:** 2023-06-19

**Authors:** Mohamad Maghnie, Paolo Bruzzi, Giorgio Casilli, Dario Lidonnici, Gioacchino Scarano

**Affiliations:** ^1^ Paediatric Clinic and Endocrinology, IRCCS Istituto Giannina Gaslini, Genova, Italy; ^2^ Department of Neuroscience, Rehabilitation, Ophthalmology, Genetics, Maternal and Child Health, University of Genova, Genova, Italy; ^3^ Clinical Epidemiology Unit, Ospedale Policlinico San Martino, IRCCS, Genova, Italy; ^4^ PharmaLex Italy SpA, Milan, Italy; ^5^ Medical Genetics Unit, AORN San Pio, Benevento, Italy

**Keywords:** achondroplasia, consensus, delphi panel, real-world, patient management, multidisciplinary team

## Abstract

**Background:**

Achondroplasia is a rare genetic disorder caused by a mutation in the *FGFR3* gene, leading to skeletal changes and other systemic complications that greatly impact the patient's quality of life. There currently are differences in achondroplasia patients' management among countries and centers within the same country.

**Method:**

A group of Italian experts discussed the best practice and the current unmet needs in the management of patients with achondroplasia though a two-round Delphi panel, between September and November 2022. The Delphi survey consisted of 32 questions covering organizational aspects, diagnosis and follow-up, and management of achondroplasia patient, and was shared among 54 experts from 25 different centers in Italy. The consensus was determined on the basis of the percentage of agreement or disagreement to each statement on a 5-point Likert scale.

**Results:**

Pediatricians (including specialists in pediatrics, medical genetics, and pediatric endocrinology) orthopedics and medical geneticists were the most represented specialists accounting for 64%, 9% and 9% of participants, respectively. The panel highlighted the need for standardized procedures to identify reference centers, the crucial role of multidisciplinary team, and effective communication among centers (Hub and Spoke model) as the essential organizational features; the importance of genetic counseling, presence of a psychologist, and clear communication during prenatal diagnosis as main points for diagnosis; early intervention by different specialists, personalized care, and promotion of a healthy lifestyle as major points for patient management.

**Conclusion:**

To ensure an adequate continuity of care over the whole lifespan of a patient with achondroplasia a shared model for patient management is suggested by Italian specialists.

## 1. Introduction

Achondroplasia is a rare genetic disorder caused by a mutation in the fibroblast growth factor receptor 3 (*FGFR3*) gene, which leads to a gain-of-function of the *FGFR3* gene, a crucial regulator of bone development that physiologically inhibits chondrocyte growth (1). Consequently, affected patients present a variety of skeletal changes such as short stature, short limbs, macrocephaly, frontal bossing, small chest, and hypotonia, in addition to several complications that develop over time, such as lumbar spinal stenosis, orthopedic issues, neurologic risk, obstructive apnea, hearing dysfunction, and obesity (2, 3). Postnatal diagnosis is straightforward, while prenatal diagnosis is challenging and can be confirmed in approximately 66.6% of the patients (4, 5).

Achondroplasia is the most common rhizomelic chondrodysplasia, its prevalence in Europe is reported to be 3.72 per 100,000 persons, according to a study based on the data from 28 EUROCAT registries, from 1991 to 2015 (6). The management of this condition is demanding and involves many pediatric specialists: thus, the multidisciplinary approach represents the best modality to optimize the care and follow-up of these patients (7). Furthermore, due to the peculiar clinical manifestations and associated comorbidities, patients with achondroplasia may experience a reduced quality of life (QoL) with lower self-esteem, difficulties in social functioning, and disadvantages in daily life activities (8). For these reasons, psychological support is recommended for patients and their families (7).

Limb lengthening surgery was the only therapeutic option to achieve significant adult height in patients with achondroplasia until recently since it can improve both height gain and function, leading to an improvement in QoL. To this end, the international consensus recommends a proper assessment of the patient by a multidisciplinary team that takes into account functional, physical, and psychosocial outcomes before proceeding with surgery (7).

Despite the recently published international consensus (4, 7, 9) and the debate on the best management of patients with achondroplasia, there are several differences in the approach to the management and follow-up of these patients among countries and centers of the same country.

Given the absence of clear indications to be followed for the management of patients with achondroplasia throughout their lifetime, a group of Italian experts was involved in a Delphi panel to gather more information on the organization of the centers dedicated to the management of these patients and to highlight best practices and current unmet needs. Notably living with achondroplasia on a daily basis, families face life-threatening conditions such as sleep-disordered breathing and foramen magnum compression; these are the first needs that must be identified, screened and treated surgically. Ear, nose and throat problems with obstructive sleep apnea are another burden. Additionally, children with achondroplasia are prone to obesity and develop orthopedic complications. Another burden is the functionality and independence of daily life which is significantly affected in children with achondroplasia. These unmet and other needs require awareness and some standardization processes at the national level and are the goals of this study. We report here the result of this consensus.

## 2. Methods

This study was a nationwide survey of specialists' practices regarding the care of patients with achondroplasia. A two-round Delphi panel (10, 11) was drafted by MA Provider, an Italian healthcare consultancy company, with the help of a facilitator, to gather information about the diagnosis and management of patients with achondroplasia. The facilitator is a neutral third-party who clarifies questions or issues that arise during the discussion and ensure that the process runs smoothly, creating a structured and collaborative environment so that members of the panel can share their opinions without being influenced by others.

A multidisciplinary steering committee with accredited expertise in achondroplasia, including a pediatric endocrinologist, a medical geneticist, and an expert in the Delphi method, met in June 2022 to prioritize the issues to be addressed based on their personal experience, the published guidelines, and more recent scientific literature. The experts produced a survey of 32 questions covering three topics: (1) organizational aspects (Supplementary Material statements 1–11), (2) specific items in the diagnosis and follow-up of patients with achondroplasia (Supplementary Material, statements 12–14), and (3) management of achondroplasia (Supplementary Material, statements 15–32). The thirty-two questions were administered in a web-based survey to an extended panel of specialists involved in the care of patients with achondroplasia.

The Delphi technique allows the participants to anonymously express their agreement or disagreement with a series of statements regarding the topic of interest based on their personal experience. The two rounds of the Delphi were organized between September and November 2022, and participants were given a response deadline of 3 weeks, from September 23, 2022, to October 16, 2022, for the first round and of 3 weeks, from October 26, 2022, to November 13, 2022, for the second round.

The outcome of the first round was analyzed by the facilitator/expert in the Delphi method, and the results were reviewed by the steering committee, which revised some statements that presented syntax/communication issues. The latter were evaluated by the steering committee and re-proposed with an explicative text during the second round of Delphi. Between the first and the second round, the time needed for data analysis and preparation of the materials was reduced to a minimum (about two/three weeks) to decrease possible dropouts. The answer to each statement was mandatory; experts who did not participate in the first round were not further involved in the second round. The analysis of the results from the second round was finally shared and discussed with all the participants in a virtual meeting. A consensus was obtained after a review of the analysis and a discussion of the results.

### 2.1. Participants

The steering committee included two physicians (pediatric endocrinologist and medical geneticist) with proven extensive experience (>20 years) in the management of patients with achondroplasia, while the facilitator was a methodologist with experience in the Delphi process development. The facilitator's duties also included coordinating communication between the steering committee and the expert group, collecting the results of each round of the survey, and supporting MA Provider in data analysis.

The members of the expert panel were defined by the steering committee, which selected clinicians with proven experience in the field of achondroplasia.

### 2.2. Definition of the consensus

The level of agreement (or disagreement) with each statement was indicated by a 5-point Likert scale (12), where a value of 1 corresponded to “Strongly disagree”, a value of 2 to “Disagree “, a value of 3 to “Quite in agreement”, a value of 4 to “In agreement”, and value of 5 to “Fully agree”.

According to the literature (13), the consensus for each statement is reached for a percentage ≥70% for Likert 4–5 values and a percentage ≤30% in the range of Likert 1–2 values. On the other hand, consensus on disagreement is reached in case a statement shows a percentage ≥70% in the Likert score range 1–2 and a percentage ≤30% in the Likert score range 4–5.

### 2.3. Ethics approval and consent to participate

The questionnaire complies with Italian data protection laws and does not require ethics committee approval or clinical trial registration, as the questionnaire did not include sensitive data.

### 2.4. Data analysis

Agreement (or disagreement) was expressed for each round by descriptive statistics (modal, median and interquartile range) after analysis with Microsoft® Excel® (2019), according to the 5-point Likert scale. Regardless of the result obtained at the end of the first round, all statements were subjected to re-evaluation by the expert panel also in the second round.

The datasets used and/or analyzed during the present study are available from the corresponding author upon request.

## 3. Results

**Figure 1:**
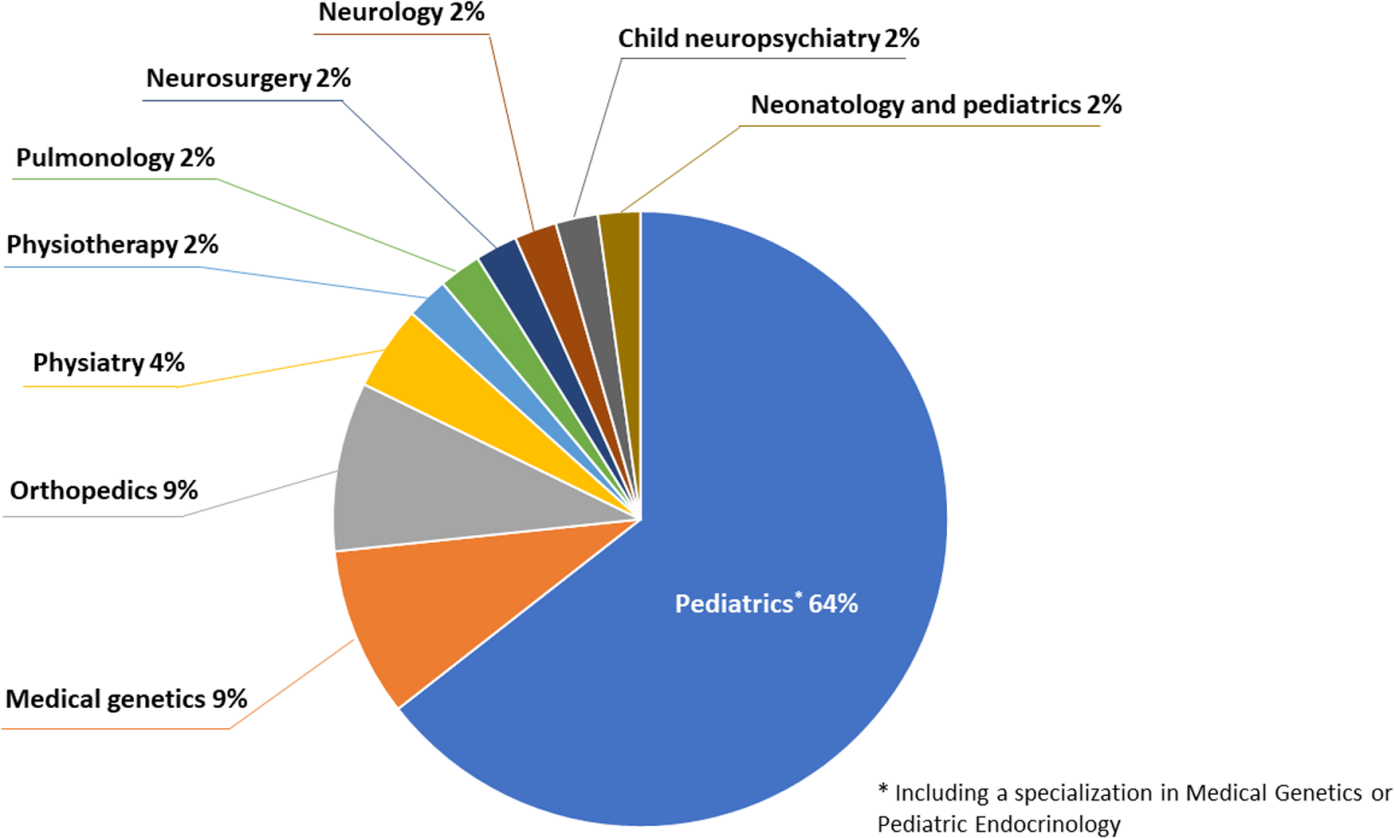
Composition of the expert panel by medical specialty.

Fifty-four experts from Italy, working in approximately 25 different centers throughout the country, were initially involved in the project and received the first set of statements. Forty-five participants (83.3%) responded to the first round, and 40 (74.0%) to the second round. The panel of experts was heterogeneous: the majority were pediatricians (including specialists in pediatrics and medical genetics, and in pediatric endocrinology) (64%) and orthopedics and medical geneticists (both 9%). The distribution of expert specialties involved in the first round is shown in Figure 1.

### 3.1. Delphi panel

#### 3.1.1. Organizational aspects

The results of statements 1 through 11, related to the organizational aspects of the centers are summarized in Table 1.

**Table 1:** Percentages of agreement with the statements focused on organizational aspects and patient follow-up, proposed in the second round of the Delphi panel.

Item	Summary of the statement	% Agreement (*n* = 40)	Mean (±*σ*) value on the Likert scale
1	It would be useful to create an interregional network on the model “Hub and Spoke” for the diagnosis and treatment of patients with achondroplasia	95	4.05 (0.78)
2	It is appropriate to identify reference centers at national level through specific qualitative-quantitative, structural, technological and process standards	97.5	4.6 (0.64)
3	It is appropriate that at the regional level, the specialized proximity centers can guarantee patients with achondroplasia an effective follow-up, to reduce the patient's discomfort and expenses	92.5	4.5 (0.72)
4	Experienced professionals are needed in specialized proximity centers with the aim of guaranteeing adequate continuity of care	85.0	4.3 (0.72)
5	Hub centers should have a multidisciplinary team with multi-specialist skills to follow a complex case study	97.5	4.7 (0.61)
6	The specialized proximity centers should identify the complications/comorbidities to optimize the care burden	90.0	4.4 (0.84)
7	The specialized proximity centers should communicate and collaborate efficiently with the Hub to optimize the management process	97.5	4.7 (0.62)
8	In the Hub and Spoke model, the role of patient organization is necessary to connect centers and families, supporting the continuity of care between specialized proximity and reference centers	80.0	4.2 (0.88)
9	It is desirable that the Hub centers coordinate the management of the adult patients with achondroplasia by providing personalized follow-up	75.0	4.0 (0.97)
10	A “health passport”, reporting the medical history of patients with achondroplasia would be useful to improve the management between different specialized centers	82.5	4.3 (0.76)
11	It is advisable to standardize the transition from pediatrics to another adult specialist, experienced in the treatment of rare diseases	95.0	4.7 (0.66)

All items achieved ≥70% expert agreement. The highest agreement (97.5%) was reached for the statements expressing the need for the recognition of standardized procedures to identify appropriate reference centers (Hubs) for the treatment of patients, the need for a multidisciplinary team within a specialized reference center, and the need for effective communication and collaboration of local care centers with the Hub for patient management optimization. Hub centers should be identified on the basis of their ability to provide complete assistance to patients (presence of a multidisciplinary team, appropriate clinical expertise and instruments for patients' evaluation). Many of the centers involved in this study are members of ERN Bond, ERN ENDO or both.

The lowest percentages of agreement were indicated for the statement defining the need for personalized follow-up of adult patients by referral centers (75%) and for the one assessing the involvement of the patient advocacy group in the continuity of care (80%).

#### 3.1.2. Specific items in the diagnosis and follow-up of patients with achondroplasia

The second group of statements, related to the diagnosis of achondroplasia, confirmed that the multidisciplinary approach is internationally recognized as the gold standard for both the diagnosis and the management of the disease (7). Table 2 reports the percentage of agreement of the second round of Delphi concerning the items related to the diagnosis (statements 12 through 14).

**Table 2:** Percentages of agreement to the statements focusing on specific items in the diagnosis and counseling of patients with achondroplasia, proposed in the second round of the Delphi panel.

Item	Summary of the statement	% Agreement (*n* = 40)	Mean (±σ) value on the Likert scale
12	Patients with achondroplasia or those who have an affected partner should be offered the opportunity to genetic counseling, starting from adolescence	95.0	4.7 (0.56)
13	Should a multidisciplinary team be available, the psychologist must support the referring clinician, starting from the communication of the diagnosis to the patients and their family	95.0	4.6 (0.5)
14	If a diagnostic suspicion is raised in the prenatal phase, adequate communication by the specialist is advisable to the parents regarding the confirmation of the diagnosis in order to avoid erroneous interpretations	97.5	4.8 (0.45)

All three items reached a high level of agreement (95%–97.5%), highlighting the recognized importance of genetic counseling for young patients with achondroplasia, the need for the involvement of a psychologist with the multidisciplinary team, and the need for clear communication between the specialist and the parents during prenatal diagnosis.

#### 3.1.3. Management of patients with achondroplasia

Table 3 summarizes the experts' opinion, after the second round of the Delphi panel, on the third group of statements (statements 15 through 32), which explore the management of patients with achondroplasia. The results highlighted that a multidisciplinary approach is essential (7); moreover, the results underline also the required characteristics of the team.

**Table 3:** Percentages of agreement with the statements focusing on the management of achondroplasia, proposed in the second round of the Delphi panel.

Item	Summary of the statement	% Agreement (*n* = 40)	Mean (±σ) value on the Likert scale
15	An ideal multidisciplinary team should consist of a health coordinator, (pediatric geneticist or pediatric endocrinologist), with support from an orthopedist, neurosurgeon, neurologist, neuroradiologist, psychologist, pulmonologist, ENT specialist, orthodontist and maxillofacial surgeon	95.0	4.6 (0.84)
16	The ENT specialist should be involved in the management of the patient early in life, as recurrent and chronic otitis media is common and of early onset	97.5	4.3 (0.49)
17	Dental prevention is essential during the formation of the primary dentition and important throughout life	95.0	4.2 (0.5)
18	A multidisciplinary check-up (orthodontist, pulmonologist, ENT specialist) is recommended at 5–6 years of age for maxillary hypoplasia related to mandibular prognathism, dental malocclusion and macroglossia	92.5	4.7 (0.62)
19	A polysomnography is advisable in the presence of suspected or evident respiratory problems or, in any case, within the first year of life	97.5	4.7 (0.51)
20	In asymptomatic infants, a magnetic resonance imaging (MRI) should be performed in the first months of life to evaluate any cervical-spinal compression and the size of the foramen magnum	80.0	4.1 (0.84)
21	Careful clinical examination for the presence of any signs or symptoms of cervical-medullary compression is necessary at any medical evaluation of infants and children and requires urgent evaluation by the pediatric neurosurgeon	100.0	4.9 (0.83)
22	A neurological evaluation is recommended every 3 months in the first year and every 6 months in the second and third years of life	95.0	4.3 (0.54)
23	Psychological support in the prenatal phase and in the first years of the child's life must be essentially family oriented	97.5	4.3 (0.51)
24	From the age of 3, psychological support must be directed to the child who is becoming aware of his condition	87.5	4.1 (0.69)
25	Since obesity is also a major health issue in individuals with achondroplasia, the recommendations to practice sport and proper nutrition, and to promote a healthy lifestyle are important for mental well-being, social inclusion and prevention of obesity	95.0	4.7 (0.58)
26	Overweight and obesity are frequent among adolescents and adults, therefore it is necessary to carry out constant weight monitoring at each visit, introducing parental food education programs	100.0	4.7 (0.46)
27	Pain should be examined and monitored over time at each visit, considering its effect on mood, personal care, education, occupation, and leisure activities	97.5	4.4 (0.55)
28	The pros and cons associated with surgical limb lengthening must be adequately assessed on a case-by-case basis by the multidisciplinary team, considering the social and psychological aspects related to the child and his family	97.5	4.8 (0.46)
29	To achieve better clinical outcomes and prevent limb lengthening complications, the multidisciplinary team should include a pediatric orthopedic surgeon, anesthetist, physical therapist, and pediatrician	97.5	4.6 (0.64)
30	The health coordinator and the orthopedic surgeon, should introduce the family and the child to the possibility of limb lengthening surgery starting from 6 years of age	97.5	4.1 (0.56)
31	Even in the absence of international consensus on the ideal age for limb lengthening surgery and the fact that it can vary from case to case, the first limb lengthening surgery is not performed before the age of 6 years	95.0	4.0 (0.62)
32	There is no international/national consensus on the appropriateness and number of limb lengthening surgeries	100.0	4.1 (0.22)

Two statements reached 100% agreement: one indicating the need to pay attention to the presence of any signs or symptoms of cervicomedullary compression, which requires urgent intervention by the pediatric neurosurgeon; the second regarding the need for a dedicated nutritional and educational program for patients and their families to avoid obesity. Among the 18 statements included in this group, seven reached 97.5% of agreement, including those concerning the need for early intervention by an ear, nose, and throat (ENT) specialist for recurrent otitis, the need to investigate respiratory problems with polysomnography, the importance of prenatal psychological support for the family who received the diagnosis for the fetus and for those who received it in the neonatal period, and finally the need to monitor pain over time and to provide the necessary information regarding limb lengthening.

Five statements reached 95% of agreement, including the composition of the multidisciplinary team, the importance of preventive dental care, the timing of neurological examination in infants and children, the importance of promoting a healthy lifestyle to reduce the risk of obesity, and the necessity of providing information on the most adequate age for limb lengthening surgery. The statement proposing the need for a multidisciplinary team involving an orthodontist, maxillofacial surgeon, and other specialists, for the diagnosis of maxillary hypoplasia in 5–6-year-old patients, achieved a 92.5% agreement, while the one regarding the children's age for starting psychological support reached 87.5% agreement. Finally, the indication for the use of magnetic resonance imaging in asymptomatic patients in the first months of life obtained 80% approval.

## 4. Discussion

In this study, we report the results of a survey conducted in Italy among 45 experts in the field of achondroplasia regarding the current clinical practice. The panel included mainly pediatric endocrinologists and pediatric geneticists, with the participation of some other specialists across major subspecialty areas involved in the care of comorbidities associated with this condition. The ten different medical specialties represented in the expert panel reflect how the real world of patient-centered care with achondroplasia is established, with a multidisciplinary team essential for the management of these patients in agreement with the most recent European Achondroplasia Forum best practice recommendations (9).

This work represents the first nationwide effort to standardize care for individuals with achondroplasia across their lifespan and specialty areas, based on available evidence and combined expert experience.

The main results of the survey highlighted the importance of a Hub and Spoke organizational system and a functional network between the tertiary reference center and regional centers on diagnosis, multidisciplinary management, and lifelong assistance of people with achondroplasia. Patient management should involve specialists and some subspecialties, beginning early in life and continuing until adulthood. Patients should be assisted according to their specific needs with personalization of care to optimize their clinical outcomes and QoL.

It is necessary to make adequate psychological support available to the families and children and to provide information on limb-lengthening surgery after careful consideration among the specialists involved in patient care, in order to improve function and reduce unnecessary surgeries. In the sections below, the statements regarding each topic will be discussed along with the most relevant survey findings and supporting scientific evidence, if available.

### 4.1. Topic 1: organizational aspects

Given the organization of healthcare facilities dedicated to the management of patients with achondroplasia, the experts believe that the Hub and Spoke model is the most efficient in providing the best care. In this model, the reference centers, identified through appropriate standards at the national level, must be connected to peripheral centers through a network that mainly depends on the local organization (virtual connection among the centers to share patients and information/telemedicine and enable personal relationships between the clinicians belonging to the various centers, regular meetings, etc.).

Highly specialized centers must be considered the reference point for the expertise and equipment for the diagnosis and treatment of achondroplasia. On the other hand, the experts recognize the importance of regional centers for routine patient care that reduces the burden of frequent visits to the Hub. In this model, the patient is assisted locally, with the possibility of periodic teleconsultation and an in-person visit only once/twice a year or when necessary. In the Hub and Spoke model, according to the opinion of the panelists, the availability of expert professional figures in the specialized proximity centers is mandatory to ensure adequate continuity of care, with the presence of a “mini” multidisciplinary team, for the management of the most frequent age-dependent comorbidities associated with this condition.

The need for a Hub center to coordinate the follow-up of a young adult patient with achondroplasia is highly controversial. Although the panel agreed with the statement, 10% of the experts disagreed. Indeed, some practical aspects should be considered, such as the fact that Hub centers specialized in the management of children may not be equipped to provide the appropriate expertise for adult follow-up. To be pointed out is the fact that the opinion of international experts is not unanimous on the actual need for regular follow-up visits for adult patients (4, 7, 8, 9, 14). Indeed, the data about the natural history of the disease and the role of comorbidities in influencing the QoL of adult patients are still poorly explored (14). Reports suggest more frequent heart disease problems, neurological complications, spinal stenosis, and arthritis. However, further studies are needed to accurately evaluate the prevalence and role of such comorbidities in adult life (14–16). In our opinion, the follow-up should be personalized or should take into account the natural history of the U-shape occurrence of comorbidities reported in adults with achondroplasia. More insights about this aspect will likely come from the Lifetime Impact of Achondroplasia Study in Europe (NCT03449368), a multinational observational study monitoring patients aged 5–70 over five years at 20 centers in Europe, collecting clinical data on the history of the disease, and on the associated clinical, psychosocial and socio-economic burden, the impact on QoL and the use of healthcare resources.

The goal for adult patients who are independent and don't present specific dysfunctions, and their correlated complications, should be to achieve good QoL. However, experts underline the need to improve the transition to adulthood in current clinical practice.

While the interaction between physicians and patients is recognized as supporting, patient advocacy groups may not be critical to the continuity of patient care, which should be primarily the responsibility of the healthcare system.

### 4.2. Topic 2. Specific items in the diagnosis and counseling of patients with achondroplasia

The participants' responses to the three items on the diagnosis and on the time and modality for counseling management, confirm the importance of the quality of communication, and the timing of the discussions with patients (prenatal for those with an affected partner or in adolescence for the patients with achondroplasia). In particular, adolescents with achondroplasia should be offered the opportunity to discuss inheritance patterns, preconception options, and other related issues. Furthermore, the participants agreed on the need for psychological support during the counseling since it allows the patient and the family to better understand the implications of the condition. Patients and families should be aware that achondroplasia is transmitted in an autosomal dominant manner and is caused by genetic alterations (pathogenic missense variants in *FGFR3*) occurring *de novo* in 80% of individuals with achondroplasia, while 10%–20% of the cases inherited the mutation with 100% penetrance from an affected parent (7).

### 4.3. Topic 3. Management of patients with achondroplasia

The major focuses concerning the management of patients with achondroplasia concerned some aspects of multidisciplinarity, the role of MRI, obesity, and limb lengthening.

There was a general consensus towards the multidisciplinary model of care as a way to improve the outcomes of patients with achondroplasia, a complex multisystem condition leading to many impairments requiring intense interdisciplinary cooperation between clinicians, geneticists, surgeons, and scientists from many disciplines. The panelist agreed that the multidisciplinary team should include a health coordinator, a pediatric geneticist or endocrinologist, as well as an orthopedic, neurosurgeon, neurologist, neuroradiologist, psychologist, pulmonologist, ENT specialist, orthodontist, and maxillofacial surgeon. The presence of all these specialists is also recommended by the international consensus (7), although the availability of the psychologist as a permanent member of the team may be problematic in the Italian setting due to a lack of resources; the presence of a physiatrist is also suggested. The multidisciplinary team and the health coordinator are central to patient management. However, throughout the patient's lifespan, one of the specialists may take on a lead role, depending on the specific burden and the natural history of the condition.

Despite the recognized crucial role of the multidisciplinary team, the experts underline that the specialists may not need to be present in the same healthcare facilities but should be part of the Hub and Spoke organization.

Regarding the management of cervical medullary and cervical cord compression implicated in the increased infant death rate in achondroplasia (17, 18), there was not a complete agreement among the experts about the published statement affirming that in asymptomatic infants with achondroplasia, MRI scanning should be considered during the first months of life to evaluate the cervicomedullary junction and the size of the foramen magnum (7). The relatively lower agreement on this statement could also be due to the possible difficulties existing in some centers in performing an MRI scan on a young child. Additionally, the skills required of an experienced pediatric neuroradiologist may not be available considering that the foramen magnum is smaller in children with achondroplasia.

Obesity is a major comorbidity in achondroplasia patients (19) and despite not being associated with diabetes and hypercholesterolemia (20), it can worsen other complications, such as orthopedic problems at the joints, and sleep apnea, and it can increase the risk of infections during elongation, especially on thighs. Moreover, obesity in adults increases the risk of heart-related problems as a higher incidence of cardiovascular disease has been shown in obese achondroplasia patients (15, 21, 22). The experts agreed on the need for constant weight monitoring and the promotion of a healthy lifestyle as a part of the multidisciplinary interventions.

The lengthening process affects not only the patient but his/her whole family; therefore, proper support should be made available. Should this not happen, this situation could cause distress and psychological issues to the patient, with consequences on the therapy and disease management.

This study presents some limitations: although the panel included representatives of most of the specialties dealing with the disease, some of them were underrepresented, which may have influenced the outcome of the discussion. The focus on the pediatric area is pivotal for establishing proper patient management; nevertheless, specific situations in adult patients should not be neglected. Given the underlined importance of nutritional and psychological aspects, the suggestions from experts in these fields would likely contribute to the improvement in achondroplasia management.

On the other hand, the proven clinical expertise of the panelists involved assures a precise picture of the real-world situation in achondroplasia management in Italy. This element should be carefully considered by stakeholders and decision-makers for any future organizational issue.

## 5. Conclusions

For adequate management of achondroplasia patients, from birth to adulthood, the presence of a multidisciplinary team including a pediatric geneticist or endocrinologist, medical geneticist, orthopedic, neurosurgeon, neurologist, neuroradiologist, psychologist, pulmonologist, ENT specialist, orthodontist, and maxillofacial surgeon is recommended. Based on the typical comorbidities of individuals with achondroplasia and the need for specialists, Italian experts recommend a Hub and a Spoke model to minimize the discomfort of families and offer the best available care. The Hub and Spoke model combines the expertise of the specialized center with the possibility of continuous local assistance for adult patients to ensure a lifelong good QoL. Genetic counseling is pivotal for understanding the implication of the disease, and patients and their families should be provided with psychological support.

## Data Availability

The original contributions presented in the study are included in the article/**Supplementary Material**, further inquiries can be directed to the corresponding author.
