# Supplementary material for: The management of achondroplasia in Italy: results from a Delphi panel based on real-world experience
Source: Front Pediatr. 2023 Jun 19;11:1209994. doi: 10.3389/fped.2023.1209994 (PMC10315838; doi:10.3389/fped.2023.1209994)
Supplement: Supplementary file 1 [file Table1.docx]

Supplementary Material

The management of achondroplasia in Italy: results from a Delphi panel based on real-world experience

Mohamad Maghnie, Paolo Bruzzi, Giorgio Casilli, Dario Lidonnici, Gioacchino Scarano

*** Correspondence:** Mohamad Maghnie: mohamadmaghnie@gaslini.org

# Full text statements of the Delphi panel submitted to the experts

Please indicate your agreement to the following statements on the basis on a 5-point Likert scale, as follows:

1: "Strongly disagree"

2: "Disagree "

3: "Quite in agreement"

4: "In agreement"

5: "Fully agree".

Organizational aspects

1. It would be useful to create an interregional network (Hub and Spoke model) for the correct integration between reference centers (Hub) and specialized proximity centers (Spoke) for diagnosis and treatment of achondroplasia patient.

2. It is appropriate, at national level, that the reference centers (Hubs) for the diagnosis and treatment of achondroplasia patient are identified on the basis of specific qualitative-quantitative, structural, technological and standard process.

3. It is appropriate, at the regional or inter-regional level, that the adequate specialized proximity centers (Spoke) are identified, for guaranteeing an efficient follow-up to achondroplasia patient, to reduce frequent and long journeys and significant increase in costs, a major cause of discomfort.

4. It is necessary to strengthen the potential of specialized proximity centers (Spoke) and the related territorial services with some professional figures who have experience in the field of rare diseases, including achondroplasia, with the aim of guaranteeing adequate continuity of care.

5. In the hypothetical Hub and Spoke model, the Hub centers should be equipped with a multidisciplinary team characterized by multi-specialist skills to be able to follow a complex case study.

6. In the hypothetical Hub and Spoke model, the specialized proximity centers (Spoke) should identify the complications/comorbidities of achondroplasia patient to optimize patient care and assistance (e.g., access to the reference center).

7. In the hypothetical Hub and Spoke model, the specialized proximity centers (Spoke) should communicate and collaborate efficiently with the Hub center and the related territorial services in order to optimize the management of achondroplasia patient.

8. In the Hub and Spoke model, the role of patient associations, connecting families and centers, is necessary to support the continuity of care between specialized proximity and reference centers.

9. It is desirable that the Hub centers coordinate the management of achondroplasia patient also in adulthood by providing for tailored follow-up as needed, on the basis of their clinical conditions.

10. It would be useful to implement a "health passport" which reports the clinical history of achondroplasia patient to improve their management between the various specialized centers.

11. During the lifetime of achondroplasia patient, it would be necessary to standardize the transition from pediatrics to the specialist experienced in the treatment of adult rare diseases.

Specific items in the diagnosis and follow-up of patients with achondroplasia

12. As early as adolescence, achondroplasia patients or those who have a partner with achondroplasia, should be offered the opportunity to refer to a specialist for genetic counseling on issues such as genetic transmission, preconception evaluation, and factors that may affect the safety and course of pregnancy.

13. Should a multidisciplinary team be available, the psychologist must support the referring clinician throughout the whole care pathway, starting from the communication of the diagnosis to patients and their family.

14. In case of suspicion of a diagnosis in the prenatal phase, an adequate communication to the parents by the specialist is advisable to explain the following steps and timing for diagnosis confirmation, to avoid erroneous communications and interpretations.

Management of achondroplasia

15. An ideal multidisciplinary team for the treatment and management of achondroplasia patient should include a health coordinator, such as the pediatrics (geneticist or pediatric endocrinologist), supported by other specialists such as orthopedist, neurosurgeon, neurologist, neuroradiologist, psychologist, pulmonologist, ENT specialist, orthodontist and maxillofacial surgeon.

16. Given the common early onset of otitis media (recurrent and/or chronic), the ENT specialist should be involved in the management of achondroplasia patient from his/her early life.

17. Dental prevention is important throughout the whole life span of achondroplasia patient, but it is essential during the formation of the primary dentition.

18. Given the occurrence of maxillary hypoplasia related to mandibular prognathism, dental malocclusion and macroglossia in achondroplasia children, a multidisciplinary check-up with orthodontist, pulmonologist, ENT specialist is advisable at 5-6 years of age.

19. It would be advisable to perform a polysomnography in the presence of suspected or evident respiratory problems or, in any case, within the first year of life of a child with achondroplasia.

20. In asymptomatic infants with achondroplasia, magnetic resonance imaging (MRI) aimed at assessing any cervical-spinal compression and the dimensions of the foramen magnum should be performed by the neuroradiologist within the first months of life.

21. Careful clinical examination for the presence of any signs or symptoms of cervical-medullary compression is necessary at every medical evaluation of infants and children with achondroplasia, and must be urgently evaluated by the pediatric neurosurgeon.

22. A neurological evaluation every 3 months, during the first year of life, and every 6 months during the second and third years of life, is recommended for all children with achondroplasia.

23. Psychological support in the prenatal phase and in the first years of life of the child with achondroplasia must be essentially family oriented.

24. From the age of 3, psychological support must also be directed to the child with achondroplasia, who is gradually becoming aware of his condition.

25. Obesity is a major health problem in achondroplasia. Early encouragement to practice sports combined with proper nutrition, promotes a healthy lifestyle, mental well-being, social inclusion and obesity prevention.

26. Since overweight and obesity are frequent in adolescents and adults with achondroplasia, constant monitoring of weight must be carried out at each check-up visit, and food educational programs must be suggested to the family.

27. In achondroplasia patient, pain should be examined and monitored over time at each check-up visit, considering its effect on mood, personal self-care, education, occupation, and leisure activities.

28. The pros and cons associated with surgical limb lengthening option must be adequately assessed on a case-by-case basis by the multidisciplinary team, taking into account the social and psychological aspects of each child and their family.

29. To achieve better clinical outcomes and to prevent complications due to limb lengthening surgery, the multidisciplinary team should include a pediatric orthopedic surgeon, anesthetist, physical therapist, and pediatrician.

30. The health coordinator with the orthopedic surgeon should introduce the family and the child with achondroplasia to the possibility of performing limb lengthening surgery only when the child is at least of school age (from 6 years of age).

31. The first limb lengthening surgery is usually not performed before the age of 6, although international/national consensus on the ideal age to perform such surgery is lacking and it should be evaluated case by case.

32. There is no international/national consensus on the appropriateness and number of limb lengthening surgeries.
